# Evaluation of Different DNA Vaccines against Porcine Reproductive and Respiratory Syndrome (PRRS) in Pigs

**DOI:** 10.3390/vaccines1040463

**Published:** 2013-10-18

**Authors:** Stefano Petrini, Giorgio Ramadori, Riccardo Villa, Paolo Borghetti, Elena de Angelis, Anna Maria Cantoni, Attilio Corradi, Augusto Amici, Maura Ferrari

**Affiliations:** 1 Umbria and Marche Experimental Zooprophylaxis Institute, via Gaetano Salvemini 1, Perugia 06126, Italy; 2 Genetic Immunization Unit, Department of Biology, University of Camerino, Via Camerini 1, Camerino 62032, Italy; E-Mails: giorgio.ramadori@unicam.it (G.R.); augusto.amici@unicam.it (A.A.); 3 Lombardia and Emilia-Romagna Experimental Zooprophylaxis Institute, Via Antonio Bianchi, 7/9, Brescia 25124, Italy; E-Mails: riccardo.villa@izsler.it (R.V.); maura.ferrari@izsler.it (M.F.); 4 Pathology Unit, Department of Veterinary Sciences, Faculty of Veterinary Medicine, University of Parma, Via del Taglio 10, Parma 43100, Italy; E-Mails: paolo.borghetti@unipr.it (P.B.); elena.deangelis@unipr.it (E.A.); annamaria.cantoni@unipr.it (A.M.C.); attilio.corradi@unipr.it (A.C.)

**Keywords:** pigs, DNA vaccines, PRRS, ORF4, ORF5, CpG, UbilacI, NeuL

## Abstract

In veterinary medicine, there have been different experiences with the plasmid DNA vaccination. In this area and with the hypothesis to demonstrate the effectiveness of different plasmids encoding porcine respiratory and reproductive syndrome (PRRS), five DNA vaccines against PRRS were evaluated for their innocuity and efficacy in pigs. Eighteen animals were divided into five groups which were injected with five (A, B, C, D, E) different DNA vaccines. Albeit, none of the proposed vaccines were able to protect the animals against PRRS virus. Only vaccines A and B were able to reduce the clinical signs of the infection. ELISA IgM were detected 30 days after the first vaccination in the pigs injected by Vaccine A or B. ELISA IgG were detected 90 days after the first vaccination in the pigs injected by Vaccine B or C. Neutralizing antibody were detected Post Challenge Days 61 (PCD) in all groups. In the pigs inoculated with Vaccine C, IFN-γ were detected 90 days after first vaccination, and after challenge exposure they increased. In the other groups, the IFN-γ were detected after challenge infection. Pigs injected with each of the vaccines A, B, C, D and E showed a significantly higher level of CD4^−^CD8^+^ lymphocytes (*p <* 0.001) after infection in comparison with their controls.

## 1. Introduction

Porcine reproductive and respiratory syndrome (PRRS) is the most economically relevant disease in swine herds. It is responsible for respiratory and reproductive clinical signs, but, in recent years, reproductive failure has been more prevalent in swine herds. The continuous circulation of the virus among the pig population causes severe economic loss for the swine industry.

**Figure 1:**
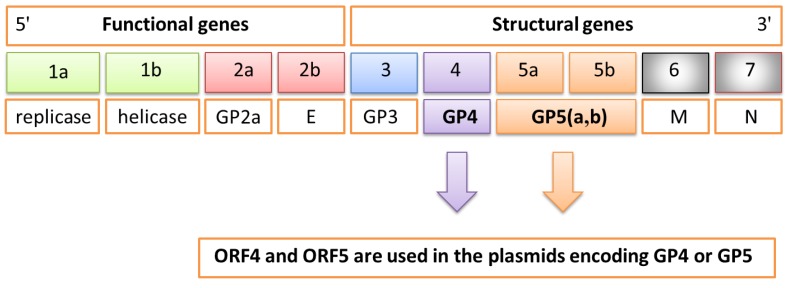
Schematic genome of porcine reproductive and respiratory syndrome virus (PRRSV) composed of 10 open reading frames (ORFs) encoding the different functional and structural proteins. In particular, ORF4 and ORF5 are used in the plasmid encoding GP4 or GP5 proteins.

The causative agent of PRRS is an enveloped virus which belongs to *Arteriviridae* family [1]. This virus contains a linear, single-stranded RNA (+) genome of 15 kb composed of 10 open reading frames (ORFs-ORF1a, ORF1b, ORF2a, ORF2b, ORF3, ORF4, ORF5a, ORF5b, ORF6, ORF7) encoding the different functional and structural viral proteins (Figure 1). In particular, the principal non-structural proteins, encoded by ORFs 1a and 1b, have replicase and helicase activities, whereas the three major structural proteins GP5, M, and N are encoded by ORFs 5, 6, and 7, respectively. The products of ORFs 2, 3, and 4 (GP2, GP3 and GP4) represent additional components of the PRRS virion. GP4 contains an immunodominant, neutralizing epitope that shows an extensive degree of variation. This fact indicates that it does not play a direct role in cell-entry or fusion processes, but that it is most probably located in close proximity to that region. Costers *et al.* indicates that accumulation of amino acids (aa) substitutions in the GP4 neutralizing epitope play a role in the inefficient PRRSV elimination from pigs with a primed anti-PRRSV neutralizing antibody response at the onset of infection [2].

The GP5 is a major envelope glycoprotein as a key PRRSV neutralization target. Monoclonal antibodies against GP5 showed neutralizing activity to the homologous strains of PRRSV. The specific sequences of neutralization epitopes in GP5 were further identified as different amino acids of the European strain (Lelystad virus, type I) or North American strain (VR-2332, type II). Also, the neutralization epitopes were defined as linear peptides. Vanhee *et al.*, 2011 have demonstrated that the antibodies specific to GP5 peptides from pigs infected with type I did not neutralize the virus [3]. Li *et al.* have demonstrated that GP5 ectodomain peptide epitopes are accessible for host antibody recognition, but are not associated with antibody-mediated virus neutralization [4].

Recently, based on the bioinformatics analysis of the gene encoding GP5, two gene fragments were amplified by PCR and designed as GP5a and GP5b, respectively. These fragments were then cloned into a plasmid vector for the production of the protein, respectively [5].

Current strategies for the control of PRRS infection include live-attenuated and inactivated vaccines. Unfortunately, these strategies of immunization are not fully successful against PRRS because they do not allow the priming of an appropriate immune response. Furthermore, reversion to virulence of the attenuated strains is of high concern as already occurred in the past. Accordingly, a high immunogenic and safe vaccine against PRRS is needed. Previous findings [6,7] demonstrated that the DNA vaccination against PRRS is at least partially successful in mice [8], suggesting that this strategy of immunization may be effective also in pigs.

The aim of this study was to evaluate the effectiveness and safety of five DNA vaccines against PRRS. The DNA-based vaccines proposed herein are plasmids encoding for ORF4 or ORF5 of PRRS. In order to increase the immune response elicited by the DNA vaccination, these plasmids were also engineered including immunostimulatory cytidine-phosphate-guanosine (CpG) motifs. Two of the vaccines also include UbiLacI, a sequence that encodes for a strong proteasomal degradation signal and that should be able to enhance the priming of a cell-mediate immunity against PRRS.

## 2. Experimental

### 2.1. Virus

The strain 2000/BS 114 L of PRRS type I was selected for this study. The virus was used at the third passage on fetal monkey kidney (MARC 145) cell cultures at a titre of 10^5.50^ TCID_50_/mL. 

### 2.2. Plasmid Vaccines

**Figure 2:**
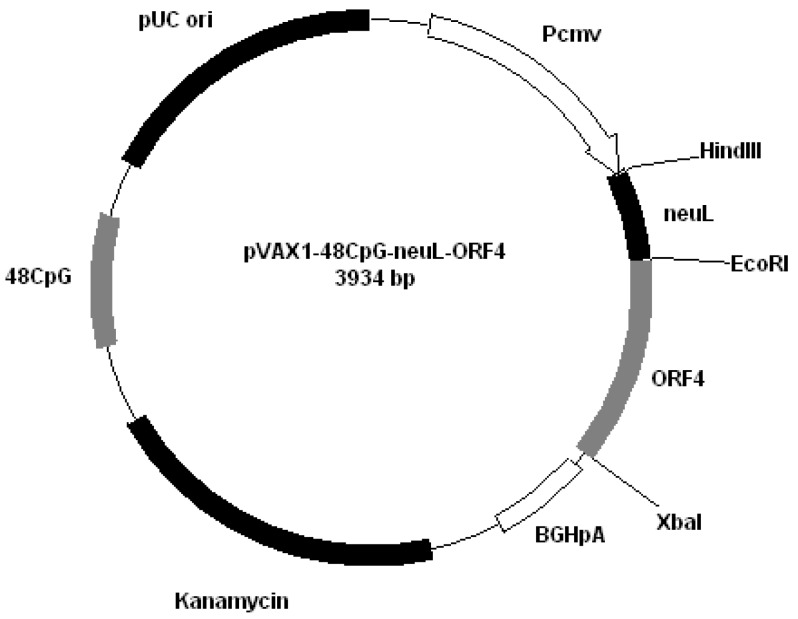
Plasmid pVAX1-48CpG-NeuL-ORF4.

**Figure 3:**
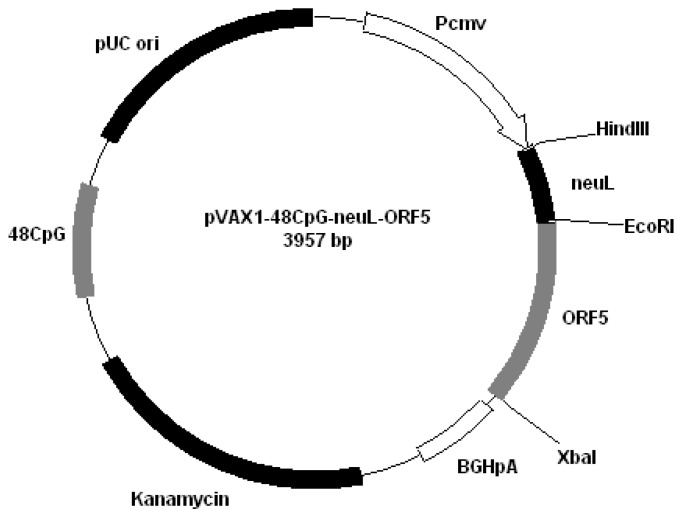
Plasmid pVAX1-48CpG-NeuL-ORF5.

**Figure 4:**
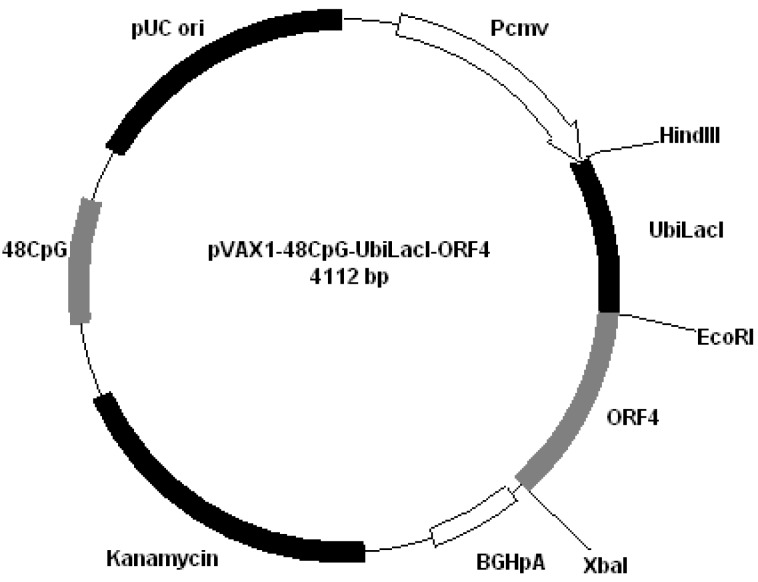
Plasmid pVAX1-48CpG-UbilacI-ORF4.

**Figure 5:**
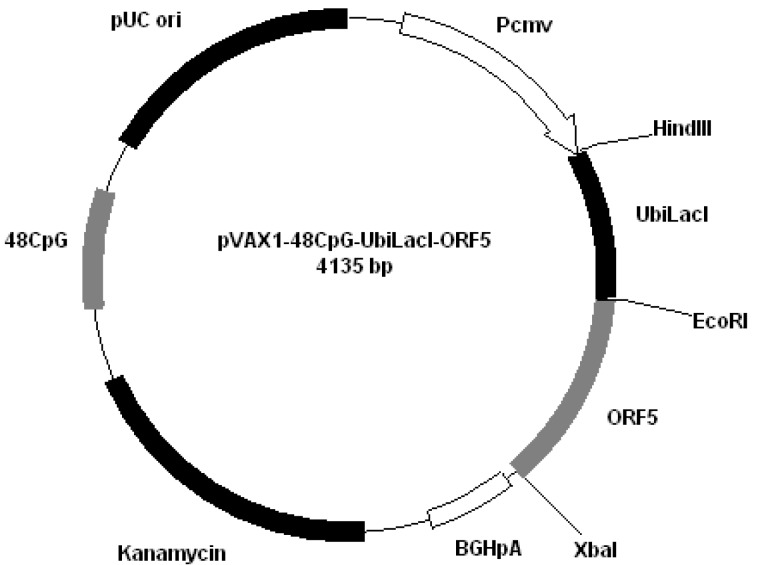
Plasmid pVAX1-48CpG-UbilacI-ORF5.

All plasmids derived from pVAX1 (Invitrogen, San Diego, CA, USA). Plasmids were constructed by cloning PRRS genes encoding GP4 and GP5 into different plasmids: pVAX1-48CpG-NeuL-ORF4 (Figure 2); pVAX1-48CpG-NeuL-ORF5 (Figure 3); pVAX1-48CpG-UbilacI-ORF4 (Figure 4); pVAX1-48CpG-UbilacI-ORF5 (Figure 5). NeuL sequence was cloned into Eco*RI* end *Hin*d III restriction sites and encoded for strong proteic secretion signal. Therefore, NeuL should allow for the protein processing by the endoplasmatic reticulum that is a step required for a new synthesized protein to be trans-located to cellular membranes and/or be secreted. These events allow for the viral antigen produced by the plasmid to be presented to the immune system in order to stimulate an antibody response. The sequence neuL includes Her-2/neu 5'UTR. The secretion signal DNA fragment was obtained by enzymatic amplification of DNA using the pCMV-ECD-TM vector [9,10] as a template, T7 primer (5'-TAATACGACTCACTATATAGGG-3') as a sense oligonucleotide and an oligonucleotide antisense having a terminal *Eco*R I site, “neuL” antisense *Eco*R I (5'-CATGGAATTCCGCGATTCCGGGGGGCAGGA-3'). The sequence UbiLacI encodes for a signal that leads to the proteasomal degradation [11]. This sequence was generated by polymerase chain reaction (PCR) from reference sequence [12] and cloned into *Hin*d III and *Eco*R I restriction sites in line with viral sequence using the following primers:

sense 5'-GCCCAAGCTTCCGGAGCCGCAGCCGCCACCATGCAGATCTTCGTGAAGACCCTGACTGGTAAGACC-3'; antisense 5'-GCCCGAATTCTCGGGAAACCTGTGGTGCCAGCTGCATTAA-3'.

The oligo sense is designed to add Kozak sequence, necessary for good transcription in mammary cells [13]. Conjugation of antigen with ubiquitin should target the endogenously synthesized antigen to the proteasome, resulting in enhanced MHC-I presentation. 

**Figure 6:**
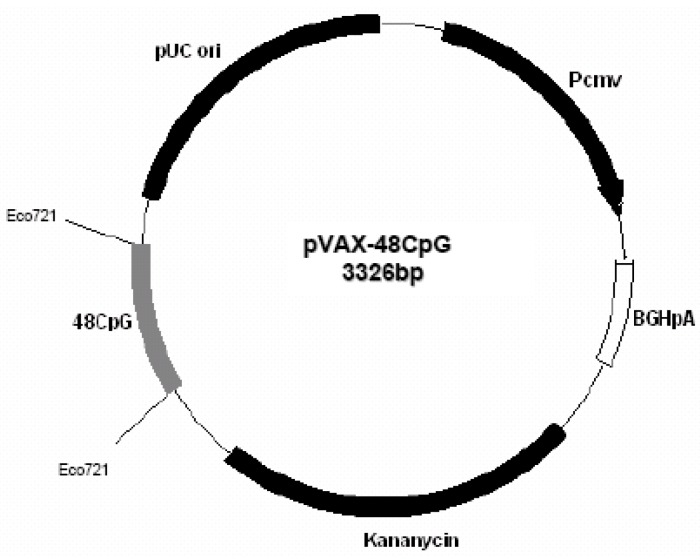
Plasmid pVAX1-48CpG.

Plasmid pVAX1-48CpG (Figure 6) was constructed by introduction in pVAX1 of specific CpG motifs based on the immunostimulatory sequence from ODN 2135 [13]. Briefly, two complementary oligodeoxynucleotides (*Bst*E II forw: 5'-AATTCGGTTACCTCTAGACAAACCAACCAAT-3'; *Bst*E II rew: 5'-CTAGATTGGTTGGTTGGTCTAGAGGTAACCG-3') were annealed to form a duplex containing the restriction site *Bst*E II, and then cloned between *Eco*R I and *Xb*a I sites in pCDNA3.1 (Invitrogen, San Diego, CA, USA). Another two complementary oligodeoxynucleotides were annealed to form a duplex containing 12 CpG motifs with protruding ends complementary to the restriction site *Bst*E II.

CpG*Bst*E II sense: 5'-GTTACGTCGTTTGTCGTTTTGTCGTTTCGTCGTTTGTCGTTTTGTCGTTTCGTCGTTTGTCGTTTTGTCGTTG-3';

CpG*Bst*E II antisense: 5'-GTAACCAACGACAAAACGACAAACGACGAAACGACAAAACGACAAACGACGAAACGACAAAACGACAAACGAC-3'.

Since the oligo CpG *Bst*E II sense has a C in spite of a G, it was possible to clone in succession four CpG annealed product into Bst*EII* locus. Finally, the whole sequence was modified by PCR using the primers: sense: 5'-GTGTGGTGGAATTGGGTTACGT-3'; antisense 5'-GTGCGGGCCCACTAGAGGAAACCAACG-3'; and blunt-cloned into *Eco*721 site of pVAX1. Plasmid DNA for immunization was purified from *Escherichia coli* strain DH5α using Qiagen Plasmid-Giga kits (Qiagen, Milan, Italy), resuspended at 1 mg/mL in sterile endotoxin water (Gibco BRI) and stored at −20 °C.

**Figure 7:**
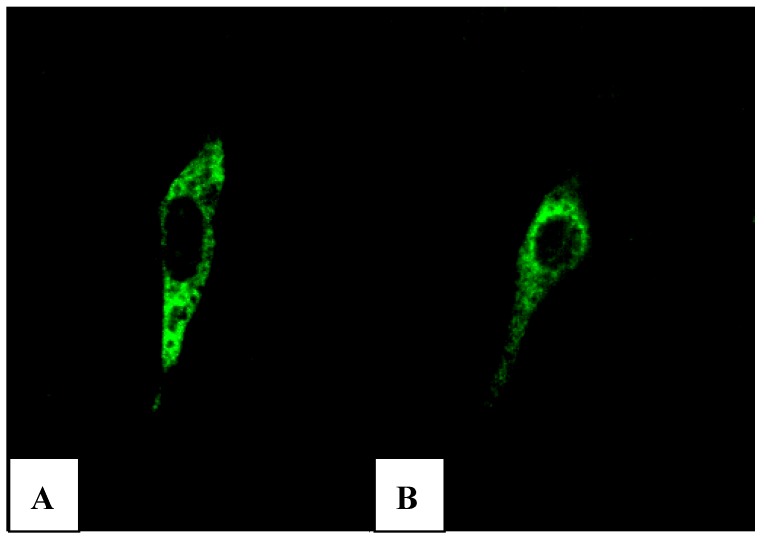
FITC immunofluorescence of mouse embryonic fibroblast cells (NIH-3T3) transfected with pVAX1-48CpG-NeuL-ORF4-Myc. (**A**) or pVAX1-48CpG-NeuL-ORF5-Myc; (**B**) after 48 h from transfection.

We cloned into the restriction site *Xba* I of pVAX1-48CpG-neuL-ORF4 and pVAX1-48CpG-neuL-ORF5 a sequence encoding for the antigenic Myc tag epitope EQKLISEEDL. This modification lead to the expression of ORF4 or ORF5 in fusion with the antigen Myc tag that is recognized by a commercial antibody FITC conjugated F2047 (Sigma-Aldrich, Milan, Italy) and then allowed us to follow the expression of ORF4 and ORF5 in mouse embryonic fibroblast cells (NIH-3T3) by confocal microscopy. Transfections of NIH-3T3 using a lipofectamine established protocol (Invitrogen, San Diego, USA) of either pVAX1-48CpG-NeuL-ORF4-Myc or pVAX1-48CpG-NeuL-ORF5-Myc led to a marked cytosolic expression of the encoded antigens (Figure 7A,B). 

### 2.3. Experimental Design

Twenty-two animals of one month of age, devoid of PRRS ELISA antibodies, were used. The pigs were housed in isolation units at Lombardia and Emilia-Romagna Experimental Zooprophylaxis Institute Brescia (Italy), and fed twice a day with a diet of concentrate and water *ad libitum*. The maintenance and experimental protocols were established according to the animal care guidelines of International Guiding Principles for Biomedical Research Involving Animals and the European Agency for the Evaluation of Medicinal Products (CVMP/IWP/07/98). The experimental design was performed after the approval of the local ethical committee.

The experimental animals were divided into seven groups (Table 1). The pigs in the first four groups each composed of four animals, were injected with the vaccine by subcutaneously (s.c.) route in the retroauricolar region; the fifth and sixth groups were injected with the plasmid with 48-CpG or only plasmid; the animals in the seventh group served as controls. Group 1 received 500 µg of pVAX1-48CpG-NeuL-ORF4 plasmid in 500 μL of 0.1 M phosphate buffer saline, (PBS) (Vaccine A); Group 2 received 500 µg of pVAX1-48CpG-NeuL-ORF5 plasmid in 500 μL of 0.1M PBS (Vaccine B); Group 3 received 500 µg of pVAX1-48CpG-UbilacI-ORF4 plasmid in 500 μL of 0.1 M PBS (Vaccine C); Group 4 received 500 µg of pVAX1-48CpG-UbilacI-ORF5 plasmid in 500 μL of 0.1 M PBS (Vaccine D); Group 5 received 500 µg of pVAX1-48CpG plasmid in 500 μL of 0.1 M PBS (Vaccine E); Pigs in group 6 received 500 µg of pVAX1 in 500 μL of 0.1 M PBS. The animals in Group 7, were used as challenge infection controls. All animals were immunized three times, at 28-day intervals. 

Ninety days following the first immunization, all animals were challenged with a virulent PRRS. The virus was given by intranasal route (i.n.) at a dose of 4 ml × 10^5.50^ TCID_50_/mL for each animal. The pigs were observed for 90 days after challenge and temperatures were taken daily for 15 days. Serum samples were taken from each pig on the day of challenge (PCD 0) and on PCD 14, 28, 61, 90. At the end of the study, the animals were killed and the target tissues (lung, mediastinic lymph-node, tonsils) were collected for histological observation.

### 2.4. Real Time PCR

Total RNA was extracted from 200 μL of each serum using RNeasy™ Mini Kit (Qiagen, Milan, Italy) by QIAcube platform (Qiagen, Milan, Italy) according to the instructions of the manufacturer, and eluted in 50 µL of RNAsi-free water. Positive serum control was previously prepared from pigs subjected to challenge infections with the PRRS type I and negative serum control was prepared from pigs free of PRRS infection. The TaqMan^®^ probe Real-Time PCR amplification was performed in the CFX96™ Real-Time System (Bio-Rad, Milan, Italy). The PRRS amplification was performed in accordance of the protocols of Revilla-Fernandez *et al.* [14].

### 2.5. Neutralization Test

Twenty-five µL of undiluted serum samples and two-fold dilutions of each were mixed with 25 µL of 100 TCID_50_ of strain 2000/BS 114 L of PRRS type I in 96-well microtitre plates (Corning Inc., Corning, New York, NY, USA). Positive serum control was previously prepared from pigs subjected to challenge infections with the PRRS type I and negative serum control was prepared from pigs free of PRRS infection. Neutralization titres were expressed as log_2_ of the highest dilution inhibiting cytopathology*.* The protocols adopted were in accordance with Yoon *et al.* [15]. 

**Table 1:** Porcine respiratory and reproductive syndrome (PRRS) DNA vaccines used in the experiment.

Group ^1^	Nu. of pigs	Vaccine identification ^2^	Type and composition	Concentration (µg/µL)	Nu. of inoculation	Inoculation route ^3^
1	4	A	pVAX1-48CpG-neuL-ORF4	500 µg/500 µL	3	s.c.
2	4	B	pVAX1-48CpG-neuL-ORF5	500 µg/500 µL	3	s.c.
3	4	C	pVAX1-48CpG-UbilacI-ORF4	500 µg/500 µL	3	s.c.
4	4	D	pVAX1-48CpG-UbilacI-ORF5	500 µg/500 µL	3	s.c.
5	2	E	pVAX1-48CpG	500 µg/500 µL	3	s.c.
6	2	Plasmid vector	pVAX1	500 µg/500 µL	3	s.c.
7	2	Challenge infection controls	NA ^4^	NA	NA	NA

^1^ All groups of pigs were housed together; ^2^ Vaccine or plasmid were administered three times, *i.e.*, 84, 56 and 28 days before challenge infection; ^3^ The vaccine or plasmid were inoculated by subcutaneous route (s.c.); ^4^ NA, not applicable

### 2.6. IgM, IgG Enzyme-Linked Immunosorbent Assay (ELISA Test)

Serum samples were used for the evaluation of immune response. Immunoglobulin M (IgM) or G IgG were evaluated by commercial ELISA tests (IgM—LSI kit, LSI, Lissieu, France; IDEXX Herdcheck—IgG PRRS kit, IDEXX Corporation, Westbrook, ME, USA). The protocols adopted were used in accordance with the instructions of the tests. 

### 2.7. IFN-γ Enzyme-Linked Immunosorbent Assay (ELISA)

IFN-γ was evaluated in serum samples by using a commercial ELISA test (Pierce, Endogen, Rockford, IL, USA). The protocol adopted was in accordance with the instructions of the test.

### 2.8. Flow Cytometry

Flow cytometry analysis was carried out according to a previous protocol [16]. Briefly, 50 μL of heparinized blood were mixed with 5 μL of the specific antibodies in a plastic tube. After 15 min of room temperature incubation in the dark, the cells were washed in PBS supplemented with 1% FCS and centrifuged for 5 min at 400 × *g*. The contaminating red cells were lysed by treatment with NH_4_Cl solution, pH 7.2, for 15 min at room temperature in the dark. The cell suspension was then washed twice in PBS supplemented with 1% FCS, centrifuged for 5 min at 400 × *g*, re-suspended in 0.5 mL in PBS supplemented with 1% FCS and finally set aside for the flow cytometry (Epics XL-MCL, Coulter). The antibodies used were as follows: anti-CD8α-FITC (Southern Biotech, Birmingham, AL, USA); anti-CD4α-R-PE (Southern, Biotech, Birmingham, AL, USA); mouse anti-pig CD8β, TCR γ/δ (VMRD Inc., Pullman, WA, USA); mouse anti-pig CD25, CD16-FITC (Serotec, Milan, Italy). 

### 2.9. Gross Pathology and Histology

Necropsies were performed after euthanasia (Tanax ®) and specimens were collected from target tissues. Specimens were fixed in buffered formalin solution 10% w/w, pH 7.4 and wax embedded (56–58 °C, Bio-Optica, Milan, Italy). Paraffin microtome sections, 5 µm thick, were stained with H&E, Van Gieson and Schiff’s reaction (PAS).

Slides were studied with conventional optical microscope (Nikon Eclipse E800) PLAN APO lens. 

### 2.10. Statistical Analysis

Positive values were statistically compared as cumulative data between treated and control groups at different times by ANOVA-Dunnett’s test. *p* < 0.05 was considered significant.

## 3. Results and Discussion

### 3.1. Clinical Response of Pigs

The test vaccines did not induce any clinical signs in immunized pigs prior to challenge at day 90. Rectal temperatures were within normal values and similar to the control values (39.0–39.5 °C). After challenge, all immunized pigs presented clinical signs which were similar to those observed in controls (Table 2). They had high fever (40.1–41.3 °C) from PCD 2 (Group 6, 7) and PCD 4 (other groups), which lasted for eight days. Inappetence, cough, dyspnoea, lethargy, were detected from PCD 1 (Groups 1 and 2), PCD 2–4 (other groups) from one to eight days. All animals recovered after three weeks following challenge. A significant difference in hyperthermia was detected in groups inoculated with vaccine A (*p* < 0.017) and vaccine B (*p* < 0.008), respectively.

**Table 2:** Clinical response of pigs immunised with experimental PRRS DNA vaccines and challenge infected with virulent PRRSV.

Group	Vaccine type and composition ^1,2^	Clinical signs after challenge infection ^3^				
		Fever ≥ 40 °C	Inappetence	Cough	Dyspnoea	Lethargy
1	A	4/8	2/5	2/2	2/2	1/2
2	B	4/8	2/4	2/2	2/1	1/2
3	C	4/8	3/6	3/3	3/3	3/4
4	D	4/8	3/5	3/2	3/2	3/3
5	E	4/8	3/6	4/6	4/5	4/4
6	Plasmid vector	2/8	2/8	2/6	2/6	2/6
7	Challenge infection controls	2/8	2/8	2/8	2/7	2/8

^1^ See Table 1 for the vaccine identification; ^2^ Vaccine or plasmid only were administered three times, *i.e.*, 84, 56 and 28 days before challenge infection; ^3^ Day of onset after challenge infection/length of period during which clinical signs were detectable.

### 3.2. Viremia

Viral RNA sequences were detected in pigs of all groups from PCD 2, 9 and 14. On PCD 20, only some animals in Groups 1, 3 and 4 were negative, while on PCD 28, 61 and 90 all pigs resulted to be devoid of virus (Table 3). No statistical significant differences were evidenced among the different groups in viremia as shown by RT-Real time PCR. 

**Table 3:** Porcine reproductive and respiratory syndrome virus (PRRSV) detection in serum samples by RT-Real time PCR from pigs, immunised with experimental DNA vaccines, and challenge infected with virulent PRRSV.

Group	Vaccine type and composition ^1,2^	RT-Real time PCR after challenge infection on days							
		0	2	9	14	20	28	61	90
1	A	0 ^3^/4	4/4	4/4	1/4	1/4	0/4	0/4	0/4
2	B	0/4	3/4	3/4	3/4	3/4	0/4	0/4	0/4
3	C	0/4	4/4	4/4	1/4	1/4	0/4	0/4	0/4
4	D	0/4	4/4	4/4	1/4	1/4	0/4	0/4	0/4
5	E	0/4	2/4	2/4	2/4	2/4	0/4	0/4	0/4
6	Plasmid vector	0/2	2/2	2/2	2/2	2/2	0/2	0/2	0/2
7	Challenge infection controls	0/2	2/2	2/2	2/2	2/2	0/2	0/2	0/2

^1^ See Table 1 for the vaccine identification; ^2^ Vaccine or plasmid only were administered three times, *i.e.*, 84, 56 and 28 days before challenge infection; ^3^ Number of pigs from which virus was recovered.

### 3.3. Neutralizing Antibody

No increase in antibody titre to PRRS was detected in the vaccinated pigs (Table 4). No seroconversion was detected in the control group inoculated with the plasmid vector. After challenge infection neutralizing antibodies evaluated on PCD 61, 90 had a titre from 1.50 log_2_ (Groups 6, 7) to 3.50 log_2_ (other groups).

**Table 4:** Serum neutralizing antibody response of pigs immunised with experimental PRRS DNA vaccines and challenge infected with virulent PRRSV.

Group	Vaccine type and composition ^1,2^	Neutralizing antibody titres to PRRSV after challenge on day ^3^				
		0	14	28	61	90
1	A	<1.00	<1.00	<1.00	3.50	3.50
2	B	<1.00	<1.00	<1.00	3.00	3.00
3	C	<1.00	<1.00	<1.00	2.00	2.00
4	D	<1.00	<1.00	<1.00	2.00	2.00
5	E	<1.00	<1.00	<1.00	2.00	2.00
6	Plasmid vector	<1.00	<1.00	<1.00	1.50	1.50
7	Challenge infection controls	<1.00	<1.00	<1.00	1.50	1.50

^1^ See Table 1 for the vaccine identification; ^2^ Vaccine or plasmid only were administered three times, *i.e.*, 84, 56 and 28 days before challenge infection; ^3^ Expressed as log_2_ of the reciprocal of the highest dilution inhibiting cytopathic effects (mean value).

### 3.4. Elisa IgM, IgG

IgM were first detected on PVD 30 in pigs injected with vaccine A or B with a mean titre of 2.01 log_2_. These titres increased to 3.07 log_2_ and 3.13 log_2_ on PCD 0, respectively, and decreased until PCD 90 with a mean titres 2.19 log_2_ and 2.10 log_2_, respectively. No IgM were detected in the other vaccinated groups as well as in the controls on PCD 0. In these animals as well as in the control group, IgM were detected only following PRRS experimental infection with mean titres of 2.78, 2.88, 2.85, 3.04, 2.91 log_2_ for pigs vaccinated with products C, D, E, plasmid vector and challenge infection controls on PCD 14. These titres did not vary significantly on PCD 28, 61, 90 (Table 5). 

**Table 5:** ELISA Immunoglobulins M (IgM) response of pigs immunised with experimental PRRS DNA vaccines and challenge infected with virulent PRRSV.

Group	Vaccine type and composition ^1,2^	ELISA IgM titres to PRRS ^3^								
		Post Vaccination Days (PVD)				Post Challenge Days (PCD)				
		0	30	61	84	0	14	28	61	90
1	A	<1.00	2.01	2.54	2.66	3.07	2.97	2.38	2.36	2.19
2	B	<1.00	2.01	2.30	2.58	3.13	2.96	2.40	2.23	2.10
3	C	<1.00	<1.00	<1.00	<1.00	<1.00	2.78	2.38	2.22	2.07
4	D	<1.00	<1.00	<1.00	<1.00	<1.00	2.88	2.47	1.99	2.17
5	E	<1.00	<1.00	<1.00	<1.00	<1.00	2.85	2.12	2.15	2.14
6	Plasmid vector	<1.00	<1.00	<1.00	<1.00	<1.00	3.04	2.34	2.44	2.27
7	Challenge infection controls	<1.00	<1.00	<1.00	<1.00	<1.00	2.91	2.63	2.64	2.60

^1^ See Table 1 for the vaccine identification; ^2^ Vaccine or plasmid only were administered three times, *i.e.*, 84, 56 and 28 days before challenge infection; ^3^ Expressed as log2 of the reciprocal of the highest serum dilution positive by ELISA (mean value).

IgG were first detected on PCD 0 in pigs inoculated with vaccines B or C with a mean titre of 1.45 log_2_ and 1.59 log_2_, respectively. These titres increased to 3.02 log_2_ and 3.03 log_2_, respectively, on PCD 61. Then, they decreased to a mean titre 2.82 log_2_ and 2.42 log_2_, respectively, until PCD 90. No IgG were detected in the other vaccinated groups as well as in the controls on PCD 0. In these animals as well as in the control group, antibodies were detected only following PRRS experimental infection with mean titres of 2.43, 2.12, 2.50, 2.55, 2.12 log_2_ for pigs vaccinated with products A, D, E, plasmid vector and challenge infection controls, respectively on PCD 14. These titres increased from 2.69 log_2_ to 3.13 log_2_ on PCD 61and decreased from 2.34 log_2_ to 2.82 log_2_ on PCD 90 (Table 6). 

### 3.5. ELISA IFN-γ test

**Figure 8:**
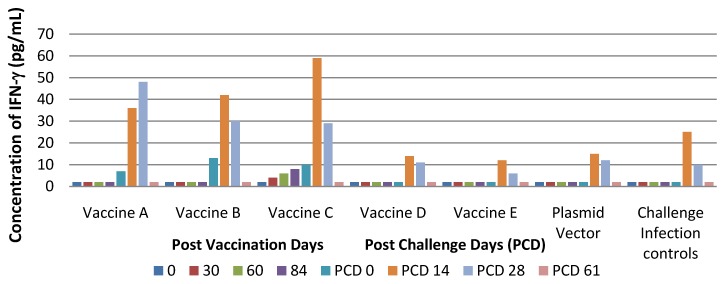
IFN-γ response (pg/mL) in pigs immunised with experimental PRRS DNA vaccines and challenge infected with virulent PRRSV.

IFN-γ were detected only in the groups of pigs inoculated with vaccines B or C on PVD 84 with a mean titre of 13 and 10 pg/mL, respectively. A further increase was detected in these animals on PCD 14 when the mean was 41 and 59 pg/mL, respectively. In the other groups immunized with Vaccines A, D, E, plasmid vector and in the challenge infection controls, IFN-γ were detected only on PCD 14, with a mean titre ranging from 12–36 pg/mL. In all groups, a decreased of IFN-γ was detected on PCD 61 with a mean titres of 2 pg/mL (Figure 8).

**Table 6:** ELISA Immunoglobulins G (IgG) response of pigs immunised with experimental PRRS DNA vaccines and challenge infected with virulent PRRSV.

Group	Vaccine type and composition ^1,2^	ELISA IgG titres to PRRSV ^3^								
		Post Vaccination Days (PVD)				Post Challenge Days (PCD)				
		0	30	61	84	0	14	28	61	90
1	A	<1.00	<1.00	<1.00	<1.00	<1.00	2.43	2.81	3.03	2.78
2	B	<1.00	<1.00	<1.00	<1.00	1.45	2.53	2.92	3.02	2.82
3	C	<1.00	<1.00	<1.00	<1.00	1.59	2.69	2.85	3.03	2.42
4	D	<1.00	<1.00	<1.00	<1.00	<1.00	2.12	2.77	2.69	2.34
5	E	<1.00	<1.00	<1.00	<1.00	<1.00	2.50	3.04	2.91	2.48
6	Plasmid vector	<1.00	<1.00	<1.00	<1.00	<1.00	2.55	3.21	3.13	2.76
7	Challenge infection controls	<1.00	<1.00	<1.00	<1.00	<1.00	2.12	2.81	2.69	2.76

^1^ See Table 1 for the vaccine identification; ^2^ Vaccine or plasmid only were administered three times, *i.e.*, 84, 56 and 28 days before challenge infection; ^3^ Expressed as log2 of the reciprocal of the highest serum dilution positive by ELISA (mean value).

### 3.6. Flow Cytometry Analysis

After challenge, a significant difference was detected in the time-related changes of CD4-CD8^+^ lymphocytes (*p <* 0.001) between the pig groups injected with the different plasmids and the controls. A higher difference was observed also on PCD 14 and 28 for γ/δ (*p <* 0.037) and CD16 (*p <* 0.0001) (data not shown). 

### 3.7. Gross Pathology and Histology

Necropsy was conducted on PCD 90. Gross pathology related to PRRS infection was observed in vaccinated and control pigs. Histopathology lymphoid organs were characterized by lymphocytic hyperplasia in infected pigs. Moreover, mononuclear parenchyma infiltrations were constantly observed in lungs. No histological lesions were observed in other target tissues collected in pigs of all groups.

## 4. Discussion

Viral glycoproteins of PRRS virus have been identified as the main targets for humoral and cell-mediate immune responses and they have been selected as candidate antigens in novel vaccine strategies such as DNA immunization. 

The genome of PRRS virus contains 10 open reading frames (ORFs). ORF1 encodes for viral replicase polyproteins that are immediately translated upon viral entry, and proteolytically processed by viral encoded proteinases into different non-structural proteins. ORF2a, ORF2b, ORF3, and ORF 4 encode for the structural proteins GP2a, GP2b (E), GP3 and GP4, respectively. ORFs 5–7 encode for three major structural proteins, respectively, *i.e.*, the envelope glycoprotein GP5, the non-glycosylated membrane protein (M) and the nucleocapsid protein (N). Immunization protocols against GP4, GP5, M, N proteins have already been tested with the aim to evaluate their safety and efficacy in miceand pigs [7,17].

Currently, the control of PRRS infections is performed by using two types of commercial PRRS vaccines based on the use of killed-virus (KV) vaccine or modified-live virus (MLV) vaccine. It was proven that KV vaccine can stimulate partial immune protection to PRRSV [18], while PRRS MLV vaccines seems to be more efficacious for protection against clinical signs induced by homologous infection; however, PRRS MLV vaccines have the disadvantage to revert to virulence [19,20,21,22]. With the aim to achieve the above mentioned, a new generation of PRRS vaccines is being explored and in recent years DNA vaccines have been able to induce effective humoral and cell-mediated immune responses in different animal models [4,5,6,7,23,24,25]. However, most viral DNA vaccines are designed to express only one antigen and, thus, their efficacy is lower compared to conventional vaccines [26,27]. Different strategies have been performed to improve DNA vaccines such as the choice of vector and target protein as well as the use of an adjuvant or co-immunogen [27,28,29]. 

The pVAX1 plasmid used in this study was selected due to its small size that provides a very high level of protein expression, thus minimizing extraneous genetic elements. This strategy appeared to be successful as the expression of proteins from different plasmids in NIH-3T3 cells was confirmed by immunofluorescence, clearly demonstrating the ability of the different genes to be expressed *in vitro*. 

In order to increase the immunogenicity of DNA vaccines, we used novel adjuvant approaches *i.e.*, the incorporation on the DNA vaccines of CpG oligodeoxynucleotides (ODN) [23]. In this study, a plasmid (pVAX-48CpG) was constructed containing 48 copies of the CpG hexamer (GTCGTT), organized in the same way as the ODN 2135 [24]. This vector was used to express the GP4 or GP5 of PRRSV (Vaccines A and B).

In Vaccines C and D, an additional sequence (UbilacI) was included in order to enhance the cell response to GP4 or GP5. UbilacI encodes for a proteasome-dependent degradation signal that mediates intracellular protein degradation and the production of peptides for antigen presentation via MHC class I. Hence, the proteasomal degradation of GP4 and GP5 should increase the number of peptides available for MHC-I binding, which may enhance the cell-mediated immune response to the vaccine antigens. 

The experimental infections conducted showed that PRRS DNA vaccines in our study did not protect pigs against infection with virulent PRRS but that they were able only to reduce clinical signs. In particular, pigs treated with Vaccines A and B developed milder clinical signs compared to controls. In contrast, pigs of Group B cleared the virus more slowly than pigs in Groups A, C, D and E.

The results obtained in this study are in contrast with those obtained from previous studies carried out with traditional PRRS inactivated vaccines. In particular, commercial KV vaccines were not able to reduce clinical signs in vaccinated pigs and challenge infected with a homologous virus [22]. On the other hand, results obtained by Vaccines A and B are similar to those detected following the vaccination with MLV vaccines which can reduce the duration of clinical signs by up to about one week [30,31,32,33]. 

A significant increase of IgM was observed only in pigs of Groups A and B (PVD 30). In contrast, IgG were detected only in Groups B and C on the day of the challenge (PCD 0). These results are in accord with data reported by others [7], following DNA vaccination and no antibodies to GP4 and GP5 were detectable by ELISA test for a period of 12 weeks after vaccination. In contrast, both KV and MLV vaccines induce IgM and IgG response due to the presence of complete virus particlesin the vaccine [32,34].

In this study, no increase in neutralizing antibody was found in all vaccinated pigs in agreement with other authors [17] but they are in contrast to the findings of Kwang *et al.* who detected neutralizing antibodies to GP4 and GP5 at a dilution 1:8 on week 12 after vaccination [7]. Similarly, Du *et al.* demonstrated the presence of neutralizing antibodies, although at a low level, following vaccination with a plasmid which expressed in fusion form GP3 and GP5 and the titres increased by using a plasmid encoding IFN-γ and IFN-α [28]. 

On the contrary, findings of studies carried out by other authors showed the inability of the GP4 and GP5 to stimulate an immune response from the host [4]. 

GP4 and GP5 of PRRSV have been able to stimulate neutralizing antibodies, and accordingly, GP5 has been suggested as a candidate for this study [7,25,35,36,37,38]. On the contrary, Li *et al.* have demonstrated the inability of pig anti-GP5 ectodomain antibodies or GP5/M ectodomain polypeptides to inhibit infection of permissive cells, indicating that GP5 and M surface epitopes are not directly involved in virus interaction with host cells [4].

The results of this study showed that plasmid encoding GP4 or GP5 gene was not able to stimulate immune response, in contrast to what was supported by others authors [8] who demonstrated the immune efficacy of GP5 associated with cytokine as adjuvant. In particular, interleukin 15 (IL-15) can activate immunologic system via IL-2 receptor and it plays a role in generating and maintaining high activity of T cell responses to pathogens. 

Observed inefficacy to stimulate neutralizing antibodies against GP4 might be the result of hypervariability of the region encoding neutralizing epitope of GP4 [2]. Moreover, failure in inducing neutralizing antibodies following DNA vaccination could be the consequence either of a reduced transcription/translation process of the ORFs cloned sequences or of the inability of the commercial ELISA test (IDEXX) to detect antigens encoded by ORF4 and ORF5 genes [39]. 

Findings from the performed study suggest that vaccine C seems to be more immunogenic than other types as indicated by viremia, lasting for a shorter time and by stimulation of cell-mediated immune response.

In contrast, NeuL sequences included in Vaccines A and B, ubiquitin sequences included in vaccine A and D and CpG motifs included in vaccine E were not able to stimulate any immune response. GP4 protein expressed with 48 CpG motifs and ubiquitin in Vaccine C, presumably due to insufficient amounts of antigen, did not stimulate B-cells and that did not produce the humoral immune response. Moreover, it is known that PRRS virus is unable to stimulate an efficient immune response as shown by the delayed appearance of neutralizing antibodies and cell-mediated immunity, following either infection with a virulent strain or vaccination [40]. According to that, a similar behaviour is expected to be detected following DNA vaccination. The unsatisfactory results of the study can be explained by the inability of the plasmid used to activate (priming) the immunological system as well as by the partial expression of virus antigens—only limited to GP4 and GP5 proteins—that can be responsible for the stimulation of an immune response at a lower level induced by a complete virus.

No DNA vaccines used in this experiment showed any residual pathogenicity for respiratory apparatus as shown by macroscopic investigations and histology. These findings could be caused by the long interval period between challenge infection and necropsy. 

## 5. Conclusions

To conclude, the results of this study indicate that vaccination of pigs with DNA vaccines expressing GP4 of PRRS combined with CpG motifs and ubiquitin sequences has been able to prime the immune system. However, this response was not able to protect the animal from virulent PRRS challenge infection as shown by the lesser severity of clinical signs and the viremia.

No DNA vaccines used in this experiment showed any residual respiratory pathogenicity as shown by anatomo-pathological investigations. These results indicated that PRRS DNA vaccines expressing GP4 combined with CpG oligodeoxynucleotides (ODN) in the plasmid backbone could be used for priming the immune system against PRRS infection.
